# Supplementary material for: Conditional chemoconnectomics (cCCTomics) as a strategy for efficient and conditional targeting of chemical transmission
Source: eLife. 2024 Apr 30;12:RP91927. doi: 10.7554/eLife.91927 (PMC11060718; doi:10.7554/eLife.91927)
Supplement: Figure 3—source data 3. [file elife-91927-fig3-data3.zip › Figure 3 source data3 part 1.zip!/Figure 3-Source data 3-part1/1067-9-CRISPResso_Report_c33kf3u3.zip!/CRISPResso_on_1067_11/3b.Insertion_deletion_substitutions_size_hist.pdf]

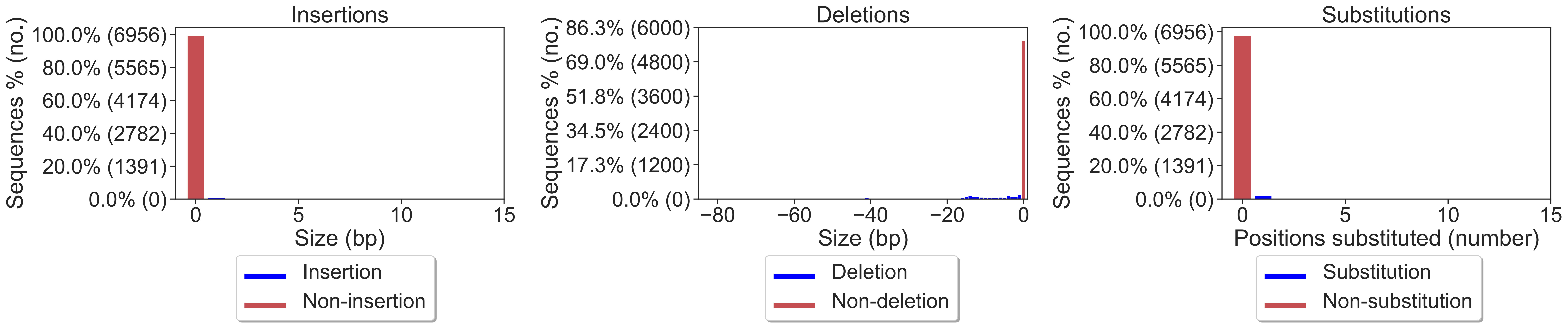
Insertions
Sequences % (no.)
100.0% (6956)
80.0% (5565)
60.0% (4174)
40.0% (2782)
20.0% (1391)
0.0% (0)
0
5
10
15
Size (bp)
Insertion
Non-insertion
Deletions
Sequences % (no.)
86.3% (6000)
69.0% (4800)
51.8% (3600)
34.5% (2400)
17.3% (1200)
0.0% (0)
−80
−60
−40
−20
0
Size (bp)
Deletion
Non-deletion
Substitutions
Sequences % (no.)
100.0% (6956)
80.0% (5565)
60.0% (4174)
40.0% (2782)
20.0% (1391)
0.0% (0)
0
5
10
15
Positions substituted (number)
Substitution
Non-substitution
